# Traditional Chinese Medicine based intervention for reducing sedentariness and improving psychological well-being in office workers: a feasibility randomized controlled trial

**DOI:** 10.3389/fpubh.2026.1744556

**Published:** 2026-02-26

**Authors:** Cong Wang, Erin Yiqing Lu, Zoey Yutong Li, Wen Sun, Jeremy Rui Chang, Waiming Cheung, Hector W. H. Tsang

**Affiliations:** 1 Department of Rehabilitation Sciences, The Hong Kong Polytechnic University, Kowloon, Hong Kong SAR, China; 2 School of Rehabilitation, Kunming Medical University, Kunming, Yunnan, China; 3 Department of Applied Social Sciences, The Hong Kong Polytechnic University, Kowloon, Hong Kong SAR, China; 4 Research Centre for Chinese Medicine Innovation, The Hong Kong Polytechnic University, Kowloon, Hong Kong SAR, China; 5 Faculty of Education, The University of Hong Kong, Pokfulam, Hong Kong SAR, China; 6 Mental Health Research Centre, The Hong Kong Polytechnic University, Kowloon, Hong Kong SAR, China

**Keywords:** office workers, psychophysiological outcomes, randomized controlled trial, sedentary behavior, TCM-based intervention

## Abstract

**Background:**

Sedentary behaviors (SBs) have been recognized as a risk factor for physical and mental health. This study primarily assessed the feasibility and acceptability of a Traditional Chinese Medicine (TCM)-based sedentariness reduction intervention to reduce SBs among office workers and explored changes in SBs and health outcomes compared to a waitlist control group.

**Study design:**

Feasibility randomized controlled trial.

**Methods:**

Thirty-eight office workers in Hong Kong, sitting over 5.5 h per workday prior to recruitment, were randomly assigned to a TCM-based intervention group (*n* = 19) or a waitlist control group (*n* = 19). The intervention included three workshops (on mind–body activities including dantian breathing, Baduanjin and stretching, acupressure related to stress management, and strategies to combat environmental factors related to SBs) over 2 weeks and another two-week period of assisted self-practice. Feasibility was based on recruitment, retention, adherence, assessment completion, adverse events, and acceptability. Intervention efficacy on sitting time at work was assessed using ActiGraph wGT3X-BT® at baseline (T1), after 4-week intervention (T2), and 4 weeks follow-up (T3). Other outcomes, including stress, quality of life, and sleep quality, were also explored.

**Results:**

The feasibility criteria of the study were all fulfilled. Rates of recruitment, retention, adherence, and assessment completion all exceeded 80%, and no adverse event was reported. Participants in the intervention group reported high satisfaction with the program. Preliminary data indicated a reduction in total sitting time in the intervention group compared to the control group, with medium effect size after intervention (mean change = −21.61 min, 95% CI: [−92.43, 4.64], Hedge’s *g* = −0.627). Among participants in the intervention group, perceived stress was reduced from baseline to T2 (mean change = −3.05, 95% CI: [−4.94, −1.16], Hedge’s *g* = −0.745). No meaningful differences were observed in other health outcomes within or between groups.

**Conclusion:**

The TCM-based sedentariness reduction intervention demonstrated feasibility, high acceptability, and preliminary efficacy in reducing SBs of office workers. A full scale randomized controlled trial is needed for conclusive findings.

**Clinical trial registration:**

https://www.chictr.org.cn/bin/home, identifier: ChiCTR2300079230.

## Introduction

According to a recent territory-wide survey in Hong Kong, above 20% of the population aged 15 or older had at least 10 sedentary hours per day (1). Notably, office-based employees were found to spend around 80% of their total working time sitting (2, 3). A substantial body of evidence indicates that high level of sedentary behaviors (SBs) exerts negative effects on numerous physical and psychological outcomes including increased risks of all-cause mortality, cardiovascular disease, metabolic disorders, and depression, independently of moderate to vigorous physical activity (4, 5). Additionally, office workers who remain sedentary for more than 3 h per day report significantly higher levels of perceived stress in comparison to those who engaged in less than 3 h of sedentary behavior (6). A systematic review of 168 studies consistently found positive associations between workplace sedentary behavior and elevated stress levels (7). The positive associations between sedentary behaviors and stress were also supported by a large-scale survey with over 34,000 adults from 6 countries (8). Therefore, it is imperative to implement interventions that alleviate sedentary office workers’ stress and enhance their wellness.

Multiple strategies have been as interventions to reduce SBs at workplace, including health education, environmental modifications (e.g., sit-to-stand workstation) (9, 10), motivational strategies (e.g., prompts, positive feedback or social competition) (11, 12) and combinations of both environmental and motivational elements (13, 14). However, Traditional Chinese Medicine (TCM) based body works were seldom applied for sedentariness reduction. TCM-based body works comprised various techniques, including dantian breathing, tuina, acupressure, cupping, gua sha, and qigong (15). These approaches were founded on the principles of qi (i.e., energy flow) and meridians, aiming to restore bodily balance through movement and stimulation of the meridian pathways (16). However, considering the implementation in offices, qigong, dantian breathing, and acupressure would be the feasible choices, given their practicality, non-invasiveness, and low space requirements. Specifically, qigong is a mind–body practice that integrates breath control, body movement, and mental focus, dantian breathing is a type of diaphragmatic breathing with mindfulness, and acupressure is the application of manual pressure—using fingers, hands, knuckles, or blunt tools—to specific acupoints on the body. All three TCM-based bodyworks have been shown as culturally relevant and easy-to-learn strategies for health promotion in Chinese population (17). The self-practice of qigong, dantian breathing, and acupressure do not require long time or large space, ease of self-practice, making them feasible alternatives to SBs in office workers. Previous studies have demonstrated various health benefits of these body works, such as enhanced cardiovascular function, immune function, and sleep quality (18–20). In addition, while psychological distress was found to be significantly associated with SBs, qigong, dantian breathing, and acupressure could significantly improve mental health (21). The TCM-based approach is likely to extend the current theories of SBs reduction, as qigong, dantian breathing, and acupressure can not only be practical alternatives to prolonged sitting, but also bring health benefits directly. They can be applied in combination with existing strategies, including health education, environmental modifications, and motivation strategies.

Therefore, it is promising to alleviate SBs in office workers in Chinese communities through a TCM-based intervention incorporating health education, environmental modifications, and motivational strategies. To our knowledge, such intervention has not been studied. It is imperative to test the feasibility of studying the intervention in Chinese office workers with a two-arm randomized controlled trial (RCT). Specifically, recruitment, retention, adherence, assessment completion, adverse event, and participants’ acceptance of the TCM-based sedentariness reduction intervention should be tested. The effects on SBs (operationalized as daily sitting time and sitting time during working hours) and physical activity can be explored. In addition, while previous interventions have been successful in reducing SBs or increasing physical activity level, the effects on health outcomes have been inconsistent (22). Hence, it is also meaningful to explore the physical and psychological benefits of the TCM-based intervention, including sleep quality, blood pressure, hand grip strength, perceived stress, general self-efficacy, and health-related quality of life.

In summary, the present study adopted the design of feasibility RCT. It was primarily to test the feasibility by conducting a two-arm RCT in Chinese office workers comparing TCM-based sedentariness reduction intervention with waitlist control. Secondarily, the study explored the effects on SBs, physical activity, and other physical and psychological outcomes. By addressing a significant gap in the literature, this study evaluates the feasibility and potential benefits of a culturally relevant TCM-based approach, which may offer an innovative and scalable solution for enhancing workplace wellness in Chinese communities.

## Materials and methods

### Study design

The study design was a two-arm feasibility RCT which adhered to the CONSORT statement for feasibility trial. All eligible participants provided informed consents.

### Participants

Potential participants were recruited through posters on the campus of The Hong Kong Polytechnic University (PolyU). To attract more potential participants, an online briefing session was provided to all the registered people to enhance their understanding of the project and confirm their intention to participate. The project team assessed their eligibility within 1 week of registration based on the following criteria.

*Inclusion criteria*: (1) Aged over 18 years; (2) full-time employees (i.e., >35 working hours per week); (3) self-report of sitting time over 5.5 h per day at work; (4) able to provide informed consent.

*Exclusion criteria*: (1) having physical or mental conditions that could interfere with their participation in the study; (2) having regular TCM-based or mind–body exercise during the month prior to recruitment.

### Intervention

The TCM-based sedentariness reduction intervention of the present study consisted of two stages.

In Stage 1, three workshops were delivered to the intervention group over 2 weeks, focused on Baduanjin (a standardized form of qigong comprising eight sequenced body movements), acupressure [12 acupoints related to stress management, including Taiyang (Ex-HN5), Yin tang (Ex-HN3), Shenting (GV24), Zan Zhu (BL2), Yuyao, Sibai (ST2), Tianshu (ST25), Qimen (LR 14), Zhangmen (LR13), Neiguan (PC6), Shenmen (HT7), Taixi (KI3)], mindfulness-based techniques and strategies on office environmental modification. Three workshops comprised one 60-min session and two 90-min sessions. The workshops were held face-to-face on the campus of PolyU. The instructors of the workshops were psychologists and occupational therapists with training and expertise in mental health and TCM-based bodywork.

Stage 2 was another two-week period of facilitated self-practice, and the project team offered regular informational, motivational, and logistic support for participants’ use of the skills learnt during Stage 1 to reduce SBs. Specifically, the support included 1-h group-based practice sessions held every other workday during lunchtime in a hybrid mode (both face-to-face and online). Moreover, a website with demonstration videos, images, and audio materials related to content of the workshops was available to the participants. They also received health tips, reminders, and prompts at 10:30 a.m. and 3:30 p.m. on weekdays to strengthen their awareness of sedentariness reduction and encourage ongoing self-practice. Participants were encouraged to practice what they have learnt for at least 30 min a day. Details about the intervention were reported in Supplementary Table 1.

### Outcomes

#### Primary outcomes: feasibility outcomes

Feasibility indicators included participants’ recruitment rate, retention, adherence and data measurement completion rate, adverse events, and intervention acceptability. The details were shown in Table 1.

**Table 1:** Overview of intervention feasibility outcomes.

Outcome	Description	Evaluation method	Evaluation timepoints
Recruitment rate	The team expected to complete recruitment within 9 weeks. A weekly average of at least five participants enrolled would indicate the feasibility of recruitment.	Recruitment logs	Baseline
Retention rate	Retention rates were monitored by the team during the first week and throughout the 4-week program. Feasibility would be demonstrated with retention rates exceeding 90% in the first week and over 80% across the entire 4-week program.	Retention logs	Throughout intervention
Adherence rate	Adherence was assessed by tracking participant attendance at three workshop sessions, six group practice sessions, and compliance with daily reminders. It was expected that participants would attend on average 80% of the group practice sessions and 75% compliance with daily reminders	Attendance record form	4-week intervention
Assessment completion rate	An overall percentage of missing data at baseline assessment, post-assessment and follow-up assessment would be calculated to understand the feasibility of assessing sedentary behavior and psychophysiological outcomes. The percentage of missing data was expected to be less than 20% at each assessment time point.	Completion and usable data logs, ActiGraph diary and analysis	Baseline, post and follow-up
Adverse events	Adverse events occurring during the implementation of the project would be systematically recorded and analyzed. It was expected that any adverse events could be explained by the deviation from the research protocol.	Self-report	Throughout intervention
Intervention acceptability	Participants’ acceptability and satisfaction were assessed via a post-program questionnaire and a brief phone interview. The questionnaire featured 16 items, covering aspects including how satisfied with the program, self-perceived benefits from the program, their intention to recommend the program to others, participants’ willingness and confidence to incorporate the skills learned into their daily work, the clarity of the instructions, and perceived usefulness of the content and frequency of daily reminder. The items are reported in Supplementary Table 2. Responses were measured using a 5-point Likert scale (1 = strongly disagree, 5 = strongly agree). An average rating of 4 or higher for each item was anticipated. In addition to the 16 items mentioned above, a comment box was included, inviting participants’ written feedback about their experience in the project. Also, during the phone interview, participants were asked about the physical and emotional benefits they gained from the intervention program, any lifestyle changes, challenges they encountered, and their recommendations for future studies. Participants’ written and verbal comments would supplement the interpretation of feasibility.	Self-designed questionnaire and brief phone interview	Post assessment

### Secondary outcomes: sedentary behaviors, psychological and physical well-being

All participants were invited to complete assessments on sedentary behaviors, physical activity, psychological distress, quality of life, sleep quality, general self-efficacy, blood pressure and hand grip strength at baseline, post-intervention (4 weeks after baseline), and 4-week follow-up (8 weeks after baseline).

#### Sedentary behaviors

Participants were advised to wear an accelerometer, the ActiGraph Wgt3X-BT (ActiGraph, Pensacola, Florida, United States), on the right side of their waist (at the intersection of a vertical line between the armpit and the knee), secured with an elastic belt, during their waking hours, excluding time spent in water-based activities, for a period of 5 workdays at baseline, post-intervention, and later at follow-up. The ActiGraph has demonstrated accuracy and responsiveness in measuring sitting time in previous studies (23–25). The participants were asked to wear the Actigraph during working days of the weeks for the assessment schedules (1) 1 week before the intervention program, (2) the week right after the completion of the program, and (3) the fifth week after the completion of the program, for the monitoring of sedentary behaviors.

An activity log was used to record the participants’ wearing of the ActiGraph and their daily sedentary time estimations. Participants were also instructed to document their daily sleep and wake times, as well as periods when the accelerometer was not worn, such as during water-based activities. This activity log, combined with daily records, was utilized to evaluate compliance with wearing the ActiGraph accelerometers. The protocol required that the ActiGraph be worn for at least 10 h each day, encompassing the full duration of the workday.

Data were downloaded from the monitor using activPAL software (version 6.16.3) and saved in 60-s epochs across awake periods. Two SB outcomes could be generated: one was the average daily total sitting time during the participant’s waking period and the other was the person’s average daily sitting time during working hours (from 9 a.m. to 6 p.m.), because all participants reported working within this timeframe. Data for a day were treated as invalid if the monitor was worn less than 10 h, recorded fewer than 500 steps, or if any single activity, such as light activity or sedentary behavior, accounted for 95% or more of the individual’s waking wear time (26). The activity log was used to verify and correct errors in the ActiGraph’s automatic wear detection. For example, if the ActiGraph failed to detect that a participant was wearing the device, but the activity log indicated otherwise, the ActiGraph record was manually corrected. Data from the ActiGraph were excluded if there was less than 1 day of valid data (27).

#### Physical activity

Participants’ physical activity was measured with the International Physical Activity Questionnaire, Taiwanese short form, a reliable self-reported tool for evaluating physical activity among adults, with correlation coefficients from 0.65 to 0.88 (28). Participants were asked to estimate the number of days they engaged in vigorous activities (e.g., swimming laps, hiking). They were also asked about moderate activities (e.g., brisk walking, gardening), as well as their walking activities. Additionally, participants provided information on how much time they had devoted to these activities during the past 7 days. Each participant’s total physical activity was determined by adding together the time spent walking and engaging in moderate and vigorous activities, with the results expressed as metabolic equivalent minutes per week (MET-minutes·wk^−1^). A higher total score reflected a greater level of physical activity (28).

#### Perceived stress

The 10-item version of the perceived stress scale (PSS-10) was utilized to assess the participants’ stress levels, and it demonstrated good reliability and validity with Cronbach’s *α*’s of 0.78–0.91 and test–retest reliability coefficients of 0.55–0.8 (29). Higher scores were correlated to more stress.

#### Health-related quality of life

The 36-Item Short Form Survey (SF-36) was used to assess the participants’ quality of life and showed good reliability and validity. Test–retest reliability for the SF-36 ranged from 0.70 to 0.93, and internal consistency with Cronbach’s alpha coefficients ranged from 0.70 to 0.95 (30).

#### Sleep quality

The Pittsburgh Sleep Quality Index (PSQI) was developed in 1989 by Buysee et al (42). The PSQI consists of 10 self-assessed sleep questions covering seven factors: subjective sleep quality, sleep latency, sleep duration, sleep efficiency, sleep disturbance, use of sleeping medication, and daytime dysfunction. The total score of each scale ranges from 0 to 21, and the higher the score, the worse the sleep quality. The Cronbach’s *α* of the original PSQI scale was 0.83, indicating good internal consistency (31).

#### General self-efficacy

The general self-efficacy scale was used to measure each individual’s self-efficacy. A total score, on a scale of 10 to 40, can be calculated. Higher scores indicate higher perceived general self-efficacy. Internal consistency ranged from 0.76 to 0.90 (32).

#### Blood pressure and hand grip strength

Blood pressure was measured using an electronic device (OMRON, HEM-7156 T). Participants were instructed to remain seated and rest for at least 5 to 10 min before the initial measurement. Blood pressure was assessed once on the left upper arm, with the cuff positioned over the brachial artery. Systolic blood pressure (SBP) and diastolic blood pressure (DBP) were recorded in millimeters of mercury (mmHg). Hand grip strength was measured using an electronic hand dynamometer (CAMRY EH101, Sensun Weighing Apparatus Group Ltd., Guangdong, China). Participants were seated with feet flat, back supported, and elbow flexed at 90 degrees, with the forearm and wrist in a neutral position. Each hand underwent three trials, with 30 to 60 s of rest between trials to prevent fatigue. The average value (in kilograms) from the three trials for each hand was recorded.

#### Demographic information

At baseline, participants provided demographic information about age, gender, height, weight, education level, health condition, smoking status, income range, work nature, work years and office size. Height and weight were self-reported by the participants. Body mass index (BMI) was calculated using the formula: weight (kg)/height (m)^2^.

#### Sample size

As a feasibility study, sample size could not be estimated based on expected effect size of the TCM-based sedentariness reduction intervention. Instead, a practical sample of 40 participants (20 per arm) was adopted according to the recommendations by Billingham et al. (33)

### Randomization

All the eligible participants were randomly assigned to either of the two study groups:

① Intervention group: a TCM-based sedentariness reduction intervention group.

② Control group: a waitlist control group.

The project team members who were blind to the objectives of the study conducted randomization sequence using random number sequence generated by Excel. Allocation ratio was 1:1. Participants were assigned to the control group if the number was odd and to the intervention group if the number was even. Group assignment information was concealed by sealed envelope.

### Implementation

**Figure 1:**
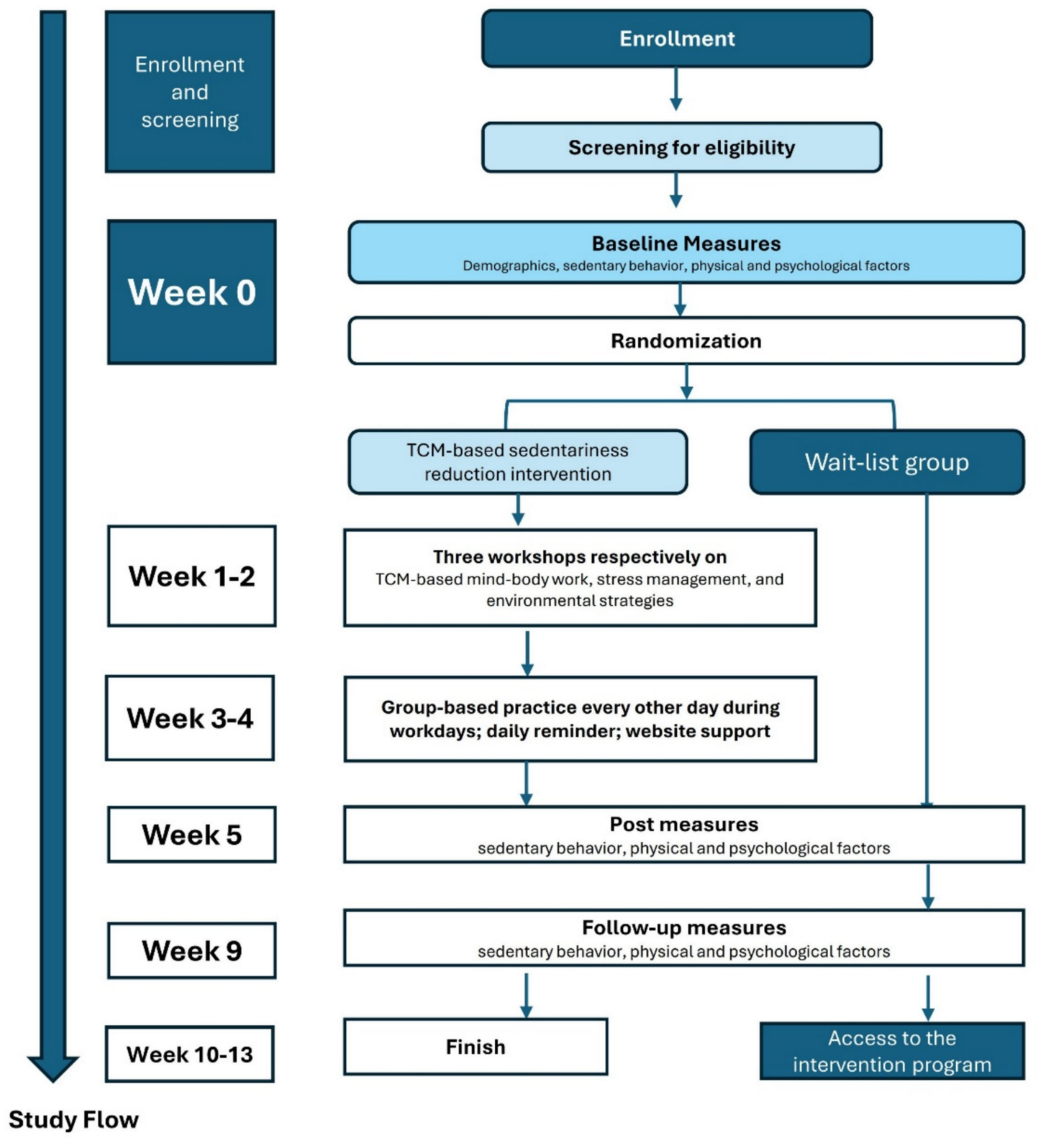
Study flow of a 4-week TCM-based sedentariness reduction intervention.

The project was implemented by the project team as shown in the author list. Recruitment and interventions were carried out by CW and EL, whereas randomization and assessment were performed by ZL and WS. All these research activities were overseen by HT. This study adhered to the principles outlined in the Declaration of Helsinki and received approval from the Chinese Clinical Trial Registry (ChiCTR2300079230). Recruitment was started after the ethical approval by the PolyU IRB was obtained (Reference number: HSEARS20230629005). Figure 1 illustrates the flow of activities that participants were engaged in. Upon the completion of all assessments, each participant received HKD 150 in cash as an appreciation of their engagement.

### Blinding

Given the design of the present study, it was very difficult to mask the group assignment and research objectives for participants. However, all the outcomes were assessed by research personnel who were blind to participants’ group assignments.

### Statistical methods

Descriptive analysis was conducted to evaluate feasibility of this study (recruitment and retention rates), the acceptability of the intervention, and participants’ overall experience with the study. The mean and standard deviation (SD) were calculated for continuous variables, while frequencies and percentages were used for categorical variables. The reliability of the self-designed questionnaire was assessed using Cronbach’s alpha coefficient. A coefficient of 0.7 or higher was considered indicative of acceptable reliability (34).

The health benefits of the TCM-based intervention for reducing daily sitting and changing secondary outcomes was explored using descriptive statistics (mean ± SD, frequency, counts and percentages). Preliminary trends in the data were explored by calculating effect sizes (Hedge’s *g*) of the between-group differences, achieved by dividing the difference in group means by the standard deviation of the pooled data. Hedge’s g values of < 0.2, 0.2 ≤ 0.5, 0.5–0.8, and > 0.8 were considered to represent very small, small, medium, and large effects, respectively (35). Paired *t*-tests were used to assess within-group differences, while independent *t*-tests were employed to evaluate between-group differences, based on the assumption of normal data distribution. Data was analyzed using SPSS v29.0 (IBM Corp., Armonk, NY, United States). The intention-to-treat (ITT) principle with last observation carried forward (LOCF) method was applied in the analysis process (36).

## Results

### Recruitment, retention, adherence, assessment completion, and adverse events

**Figure 2:**
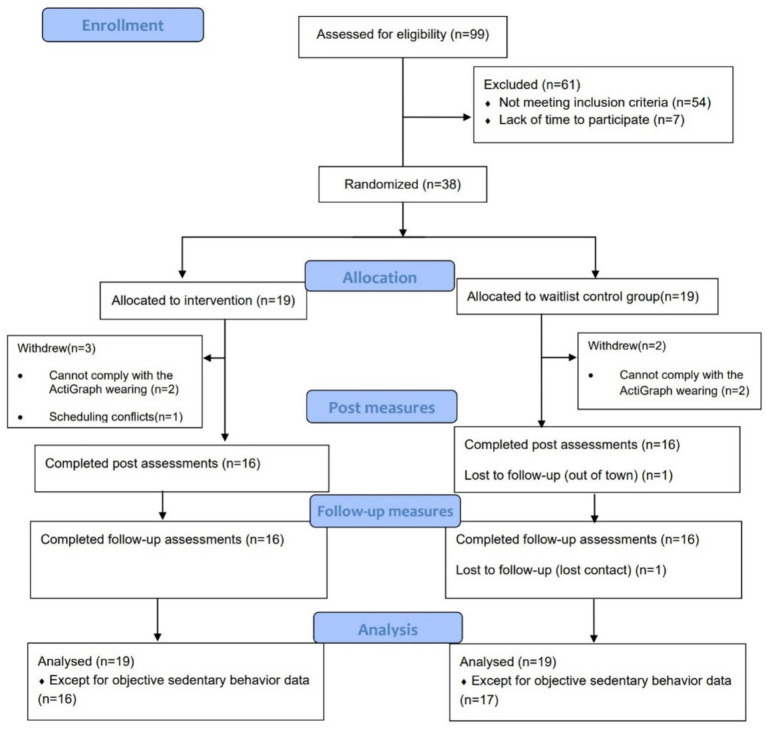
CONSORT flow diagram of the study implementation.

Over a 9-week recruitment period, 99 individuals indicated their interest in the study, and 45 were eligible to participate. Seven individuals declined to participate due to scheduling conflicts. The overall recruitment rate of individuals who were eligible was 84.44% (38/45). Thirty-eight participants were randomly assigned to either the intervention group or the control group. The retention rate after the first week was 94.74% (exceeding the expected 90%, with one participant withdrawn from the study), and 84.21% (above the anticipated 80%, with another two participants withdrawn from the study) at the end of the 4-week program in the intervention group. Reasons for withdrawal included challenges with adhering to the use of the ActiGraph (*n* = 2) and participant’s busy schedule (*n* = 1). A total of 16 participants completed the 4-week intervention, which included three workshops and six group practice sessions, with an overall attendance rate of 87.5%. After 4-week intervention, 86.84% of the participants were retained (84.21% in the intervention group and 89.47% in control group) with one participant withdrawal at 4-week follow-up in control group. No adverse event was reported during the study. In summary, participants’ recruitment rate, retention rate, adherence rate, and assessment completion rate were all in line with the expected criteria of feasibility. The CONSORT diagram of the study is shown in Figure 2.

Data collection was from December 2023 to August 2024. Completion rates for the intended primary outcome (objective measured daily sitting) were 84.21% for intervention group and 89.47% for control group at baseline. At post-intervention and 4-week follow-up, completion rates were 100 and 94.12% for the intervention and control groups, respectively. Table 2 shows the demographic information of the participants. No significant differences were found between two groups in any demographic variable.

**Table 2:** Demographic information of participants.

Characteristics	Mean (SD)/*N* (%)	Mean (SD)/*N* (%)	*χ*^2^/*t*	*p*-value
	Intervention group (n = 19)	Control group (n = 19)		
Demographics				
Age (years)	34.84 (11.19)	34.11 (11.32)	0.202	0.841
Sex			0.000	1
Male	6 (31.6%)	6 (31.6%)		
Female	13 (68.4%)	13 (68.4%)		
BMI (kg/m2)	21.89 (2.43)	22.07 (2.49)	−0.22	0.827
Smoking status			0.000	1
Current smoker	0	0		
Non-smoker	19 (100%)	19 (100%)		
Education level			2.606	0.272
Primary school	0	0		
Secondary school	2 (10.53%)	1 (5.26%)		
Tertiary education	2 (10.53%)	0		
Undergraduate and above	15 (78.95%)	18 (94.74%)		
Work nature			3.034	0.219
Clerical	4 (21.05%)	9 (47.37%)		
Professional (research, teaching, health care)	10 (52.63%)	6 (31.58%)		
Administrative	5 (26.32%)	4 (21.05%)		
Office size (n, %)			1.781	0.619
Cell office (1 person)	2 (10.5%)	1 (5.3%)		
Shared office (2–3 people)	3 (15.8%)	1 (5.3%)		
Small landscape office (4–9 people)	4 (21.05%)	6 (31.58%)		
Medium-size office (10–24)	10 (52.63%)	11 (57.89%)		
Monthly income			0.000	1
10 k-20 k	6 (31.58%)	6 (31.58%)		
20 k-40 k	11 (57.89%)	11 (57.89%)		
40 k-60 k	2 (10.5%)	2 (10.5%)		
>60 k	0	0		
Work duration			2.323	0.313
<5 years	13 (68.4%)	14 (73.68%)		
5 to 10 years	1 (5.3%)	3 (15.8%)		
>10 years	5 (26.3%)	2 (10.5%)		

### Intervention acceptability

Sixteen participants who completed the intervention expressed satisfaction with the research project, yielding a mean satisfaction score of 4.12 out of 5. The questionnaire demonstrated high reliability, with a Cronbach’s *α* of 0.928. Supplementary Table 2 shows details of the participants’ responses concerning their experiences with the study and its procedures.

After the intervention, 87.5% of participants expressed that the intervention effectively reduced their SBs, while 6.25% had neutral or dissenting views on its impact. More than 75% of participants reported being able to perform Dantian breathing, Baduanjin exercises, and mindful stretching independently by following the demonstration videos. In contrast, proficiency in performing acupressure was lower, with 56.25% of participants expressing confidence in this practice. The majority (81.25%) found the intervention materials easy to understand and considered the frequency and content of the daily reminders twice per weekday to be appropriate. However, adherence to the reminders was with 56.25% of participants consistently or frequently following them.

The interview results showed that 93.75% of participants reported improvements in both their physical health and emotional well-being, while 81.25% indicated that the program had positively impacted their lifestyle following the intervention. One participant commented, “The training served as a helpful reminder to exercise more and regulate my emotions.” Another participant noted, “Long periods of sitting and lack of physical activity previously led to tightness in my lower back muscles, but this has improved. The Baduanjin exercises are simple to learn and can be practiced regularly.” However, some participants faced difficulties, such as, “It was challenging to participate in the group exercises during lunch, as it took time away from eating.” Detailed comments on the phone interview are summarized in Supplementary Table 3. In general, the intervention was accepted by the participants.

### Participants’ baseline characteristics

Participants’ baseline characteristics are presented in Supplementary Table 4. Their total sitting time throughout the entire workday was 537.37 min for the intervention group and 547.31 min for the control group. The intervention group and the control group spent an average of 344.68 min and 351.47 min, respectively, sitting during the 9-h working time. No statistically significant differences were observed between the groups in terms of sitting time and health-related measures.

### Potential of the intervention to reduce daily sitting and improve secondary outcomes

Table 3 summarizes intervention-related changes in SBs and psychophysiological measures, following the 4-week intervention while Supplementary Table 5 presents details of the 4-week follow-up results and changes from baseline. Data on the number of valid ActiGraph wear days during the study period are provided in Supplementary Table 6. Post intervention, the intervention group reduced total daily sitting time by 21.61 min (SD = 60.45) and sitting time during working hours by 16.24 min (SD = 45.69). Conversely, the control group increased sedentary time in both measures. As a result, the intervention group demonstrated a trend of greater reduction in sedentary behavior compared to the control group, with medium effect sizes (Hedge’s *g* = 0.627 for total daily sitting time and Hedge’s *g* = 0.588 for sitting time during working hours). However, the differences in change scores between the two groups for both total sitting time and sitting time during working hours did not reach statistical significance (*p* = 0.075; *p* = 0.057, respectively). The intervention group also showed significantly more increases in physical activity relative to the control group, with a medium effect size (Hedge’s *g* = 0.705, *p* = 0.043). Furthermore, after the 4-week intervention, the intervention group experienced a significant reduction in perceived stress (*p* = 0.003), while the control group did not have significant changes in perceived stress (Hedge’s *g* = 0.333). The intervention group also showed a trend to outperform the control group in enhancing right-hand grip strength, with small effect size (Hedge’s *g* = 0.423). However, these between-group differences were not statistically significant (*p* = 0.302 and *p* = 0.192, respectively). Although the control group showed trends of greater improvements in sleep quality and the physical fitness component of the SF-36 compared to the intervention group (Hedge’s *g* = 0.546 for sleep quality and 0.402 for the physical fitness component), these differences between-group were not statistically significant (*p* = 0.213 and *p* = 0.094, respectively).

**Table 3:** Changes in sedentary behavior, physical activity, psychological, and physical well-being outcomes from baseline to post-intervention.

Outcomes	Group	Baseline (T1)	Post-intervention (T2)	Within-subject difference			Change from baseline to post-intervention	Between-group differences in change from baseline to post-intervention		
		Mean (SD)	Mean (SD)	*t*	*p*	Hedges’ *g*	Mean (SD)	*t*	*p*	Hedges’ *g*
Total sitting time (min/workday)	Intervention	537.37 (86.27)	515.66 (90.93)	1.43	0.173	0.339	−21.61 (60.45)	−1.844	0.075	−0.627
	Control	547.31 (109.22)	569.59 (70.99)	−1.226	0.238	−0.283	22.28 (74.96)			
Sitting time (min/9 h workday)	Intervention	344.68 (54.78)	328.44 (74.28)	1.422	0.176	0.337	−16.24 (45.69)	−1.976	0.057	−0.588
	Control	351.47 (56.72)	367.47 (54.56)	−1.377	0.187	−0.318	16.00 (47.91)			
IPAQ	Intervention	1529.65 (763.34)	3023.09 (1951.9)	−3.042	0.008	−0.702	1493.44 (2024.44)	2.107	0.043	0.705
	Control	2240.21 (1644.92)	2441.82 (1966.18)	−0.549	0.591	−0.127	201.62 (1514.88)			
Perceived stress	Intervention	16.32 (6.53)	13.26 (7.21)	3.392	0.003	0.745	−3.05 (3.92)	−1.048	0.302	−0.333
	Control	19.79 (6.69)	18.37 (7.91)	1.118	0.278	0.246	−1.42 (5.54)			
Hand grip (left/kg)	Intervention	24.06 (9.85)	24.59 (9.81)	−1.404	0.177	−0.308	0.54 (1.67)	−0.025	0.98	−0.008
	Control	22.78 (6.41)	23.34 (5.85)	−0.753	0.461	−0.166	0.56 (3.23)			
Hand grip (right/kg)	Intervention	27.05 (10.69)	27.57 (10.81)	−0.887	0.387	−0.195	0.42 (2.06)	1.331	0.192	0.423
	Control	25.31 (7.47)	23.79 (10.81)	1.093	0.289	0.240	−1.42 (5.64)			
SBP (mmHg)	Intervention	112.58 (17.37)	112.00 (17.40)	0.388	0.702	0.085	−0.58 (6.50)	0.197	0.845	0.062
	Control	112.53 (12.99)	111.37 (13.84)	0.456	0.654	0.1	−1.16 (11.06)			
DBP (mmHg)	Intervention	71.95 (10.34)	71.84 (11.62)	0.086	0.933	0.019	−0.11 (5.34)	−0.343	0.734	−0.109
	Control	70.26 (7.33)	70.79 (8.66)	−0.383	0.706	−0.084	0.53 (5.99)			
General self-efficacy	Intervention	26.74 (4.28)	26.95 (5.55)	−0.233	0.818	−0.051	0.21 (3.94)	−0.191	0.849	−0.063
	Control	25.06 (5.21)	25.94 (5.94)	−0.609	0.552	−0.144	0.44 (2.87)			
PSQI_total score	Intervention	5.89 (3.25)	5.58 (2.69)	1.242	0.23	0.273	−0.32 (1.11)	1.720	0.094	0.546
	Control	7.05 (3.03)	6.05 (2.70)	3.269	0.004	0.718	−1.00 (1.33)			
Quality of life_SF-36										
Physical component summary (PCS)_T score	Intervention	51.29 (6.98)	51.92 (6.85)	−0.643	0.528	−0.141	0.63 (4.25)	−1.267	0.213	−0.402
	Control	47.17 (7.64)	49.75 (8.10)	−2.167	0.044	−0.476	2.58 (5.18)			
Mental component summary (MCS)_T score	Intervention	45.52 (11.42)	48.07 (11.77)	−2.476	0.023	−0.544	2.54 (4.48)	0.288	0.775	0.091
	Control	38.19 (13.72)	40.06 (13.57)	−0.887	0.387	−0.195	1.87 (9.19)			

DBP, diastolic blood pressure; IPAQ, International Physical Activity Questionnaire; PSQI, Pittsburgh Sleep Quality Index Scale; SBP, systolic blood pressure.

Not all of the above effects maintained at the 4-week follow-up (see Supplementary Table 5). Regarding the sitting time during 9-h working time, the intervention group had more reduction than the control group with small effect size (Hedge’s *g* = −0.407, *p* = 0.24). Medium effect size was demonstrated in group difference in the increase of physical activity (Hedge’s *g* = 0.545, *p* = 0.113), with larger increase in the intervention group. Conversely, the control group demonstrated greater improvements in both the physical and mental components of quality of life, though these effects were small (Hedges’ *g* = −0.326 and −0.476, *p* = 0.311 and *p* = 0.143, respectively). However, none of these between-group differences reached statistical significance.

## Discussion

With the design of feasibility RCT, the present study demonstrated the feasibility of implementing and evaluating a TCM-based intervention aimed at reducing sitting time among office workers. Recruitment was successfully completed within a reasonable timeframe, with an average of 11 participants enrolling per week during the planned nine-week period—more than double our initial estimate of five per week. Retention was also robust. Throughout the 4-week intervention, the retention rate was sustained at 84.21%, exceeding the projected 80%. The intervention adherence rate reached 87.5%, meeting our target for participants to attend at least 80% of the group practice sessions. In addition, data completion rates at each assessment stage in both groups consistently exceeded 80%, again in line with our expectations. Even more importantly, no adverse events were reported by the participants. Overall, the intervention was deemed both acceptable and feasible, with regular group practice sessions, daily prompts, and website support from the research team identified as particularly valuable components.

While most participants demonstrated strong comprehension of the intervention content, showing confidence and dedication to maintaining their practice of qigong, dantian breathing, and mindfulness stretching exercises, a small subset expressed hesitancy about their ability to perform self-administered acupressure techniques post-intervention. This suggests that, in future studies focusing on self-administered acupressure, it will be important to provide additional support and motivation to encourage participants to increase their practice frequency and repetition, which may enhance their confidence and competence in these techniques.

This study extends the findings from previous studies with office workers by demonstrating that a merged intervention involving Baduanjin, stress management strategy and prompt tools, and website support has potential for reducing daily sitting in office workers (37). Encouragingly, a drop in daily sitting time was observed both during working hours, with such improvement sustained during the follow-up phase. This discrepancy might have been attributed to the high adherence rates in our study, contrasting with previous studies that reported declining adherence over time in intervention groups (37). Additionally, regular professional-led group sessions in our study allowed participants to clarify queries, master skills, and boost confidence in sustaining practice after the intervention. This likely has contributed to the sustained reduction in sedentary time observed during the follow-up phase. However, it is important to note that the differences in change scores between the two groups were not statistically significant, likely due to the small sample size. And the effect size of total daily sitting time at follow-up diminished to a small level. This trend aligns with prior research suggesting that exercise and motivational interventions may yield significant short-term effects but struggle to maintain long-term sustainability (22). Future studies focusing on exercise or motivational strategies may need to extend the duration of the intervention to foster habit formation and enhance long-term effectiveness (38, 39).

Additionally, the TCM-based intervention was effective in reducing perceived stress levels and showed a trend in improving handgrip strength within the intervention group. Although no significant differences were found between the groups for either outcome, the intervention still demonstrated potential not only to directly reduce SBs, but also counteracted the negative consequences caused by SBs (7, 40). These results were in line with recent studies highlighting the effectiveness of mindfulness for managing stress in workplace health promotion (41). It is warranted to evaluate such health benefits and examine the underlying biopsychosocial mechanism in future RCTs. However, the control group showed greater improvements in quality of life compared to the intervention group, likely due to their lower baseline SF-36 scores, which allowed for more potential improvement. Nevertheless, this difference was not statistically significant between the two groups. With larger sample size in the future, the intervention group and the control group would be statistically equivalent in quality of life at baseline which may allow researchers to evaluate the effect more validly. Furthermore, it remains unclear why the control group showed more improvement in sleep quality than the intervention group. Objective measures of sleep parameters and sleep logs to be collected in the future would be necessary to understand the effects of the TCM-based intervention on sleep quality.

### 4.1 Limitation and future directions

This study presents several limitations that require further refinement. (1) Most of the secondary outcomes relied on self-reported data, which may be too subjective and introduce recall bias. Future research should incorporate physiological measures. For example, stress markers, such as cortisol levels, can be collected to evaluate intervention’s impact on stress. (2) The participants in this study were predominantly young, healthy, female office workers, which may have limited the generalizability of our findings to those with pre-existing health conditions, those in different work environments, male office workers, and older employees. (3) The sample size in the present study was relatively small and lacked sufficient power to detect significant differences in the primary and secondary outcomes. Future research should include larger sample sizes and examine the intervention in more diverse populations to validate its broader applicability. (4) The missing data were handled by LOCF. If not, this might underestimate the variability of the data and affect the confidence interval and *p*-value, leading to potential bias. As more advanced statistical techniques can provide more reliable and less biased results, they should be considered for managing missing data in future research. (5) Moreover, the possibility of Hawthorne effects cannot be excluded, as repeated assessments and frequent interactions with researchers may have influenced participants to alter their usual behavior. This potential bias should be considered when interpreting the study findings.

## Conclusion

This study demonstrated the feasibility and acceptability of delivering a TCM-based sedentariness reduction intervention to reduce sitting in office workers and improve certain health outcomes. It is crucial to conduct a full-scale RCT to draw a conclusive finding and explore its long-term sustainability across diverse population.

## Data Availability

The raw data supporting the conclusions of this article will be made available by the authors, without undue reservation.
