# Supplementary material for: Traditional Chinese Medicine based intervention for reducing sedentariness and improving psychological well-being in office workers: a feasibility randomized controlled trial
Source: Front Public Health. 2026 Feb 26;14:1744556. doi: 10.3389/fpubh.2026.1744556 (PMC12979383; doi:10.3389/fpubh.2026.1744556)
Supplement: Supplementary file 1 [file Data_Sheet_1.docx]

**Supplementary Table 1.** Overview of the TCM-based sedentariness reduction intervention

**Supplementary Table 2** Responses regarding the study implementation within the intervention group

**Supplementary Table 3** Participants’ Feedback in the phone interview

**Supplementary Table 4** Baseline characteristics of participants

**Supplementary Table 5** Changes in sedentary behavior, physical activity, psychological and physical well-being outcomes from baseline to follow-up

**Supplementary Table 6** The number of valid ActiGraph wear days during the experimental period

**Supplementary Table 1.** Overview of the TCM-based sedentariness reduction intervention

| Stage | Week | Health promotion activities/strategies | Details |
| --- | --- | --- | --- |
| 1 | 1 | Workshop 1 (face-to-face; 90 min) | - Brief information on sedentary behaviors and their health risks - Mindfulness-based activities: breathing and stretching - Baduanjin (part 1: Form 1 to Form 5) |
|  | 2 | Workshop 2 (face-to-fac; 60 min) | - Acupressure (part 1: Technique 1 to Technique 5) - Strategies to combat environmental factors related to sedentariness |
|  |  | Workshop 3 (face-to-face; 90 min) | - Baduanjin (part 2: Form 6 to Form 8) - Acupressure (part 2: Technique 6 to Technique 10) |
| 2 | 3 & 4 | Daily reminders and health tips | - Twice reminders to reduce sedentary behaviors and daily feed of health tips about sedentary behaviors and safety notes on self-practice of qigong, acupressure, and mindfulness-based activities were send to the participants throughout WhatsApp - Participants were encouraged to practice what they have learnt for at least 30 minutes a day. |
|  |  | Six group practice sessions | - Participants engaged in group practice sessions every other workday, incorporating qigong, acupressure, and mindfulness-based activities. Each session lasted one hour, totaling six sessions over the course of the program. |
|  |  | Website support | - Website support to address sedentariness at the workplace throughout the entire study. |
|  |  | Environmental modification | - Small office decoration as a reminder of reduced sedentary behaviors - Rearrangement of office supplies |

**Qigong-Baduanjin**

| Form | Name |
| --- | --- |
| 1 | Prop Up the Sky with Both Hands to Regulate the Triple Warmer |
| 2 | Draw a Bow on Both sides like Shooting a Vulture |
| 3 | Raise Single Arm to Regulate Spleen and Stomach |
| 4 | Look Back to Treat Five Strains and Seven Impairments |
| 5 | Sway Head and Buttocks to Expel Heart-Fire |
| 6 | Pull Toes with Both Hands to Reinforce Kidney and Waist |
| 7 | Clench Fists and Look with Eyes Wide Open to Build up Strength and Stamina |
| 8 | Rise and Fall on Tiptoes to Dispel All Diseases |

**Important notes of self-acupressure**

| Basic principles of self-acupressure | - Start with the left side, then proceed to the right. - Stimulate each acupoint for one to two minutes. - Apply pressure until a mild sensation of soreness, numbness, pain, or distension is experienced. |
| --- | --- |
| Acupressure techniques used | - Apply pressing and kneading techniques using the thumb. - Press downward vertically with the thumb tip, kneading in a clockwise direction for around 20 strokes, followed by 20 strokes in an anticlockwise direction. - Ensure that fingernails are kept short. |
| Ten techniques | Technique 1: Press and knead the Taiyang Acupoint |
|  | Technique 2: Use both hands to massage in an up-and-down motion from the Yintang to the Shenting repeatedly |
|  | Technique 3: With both hands, massage from Yintang through Zanzhu, Yuyao, and then to Taiyang repeatedly |
|  | Technique 4: Press and knead the Sibai Acupoint |
|  | Technique 5: Tap the head gently with the finger |
|  | Technique 6: Press and knead the Tianshu Acupoint |
|  | Technique 7: Massage along the Liver and Gallbladder Meridians, the area covers Qimen and Zhangmen Acupoint |
|  | Technique 8: Press and knead the Neiguan Acupoint |
|  | Technique 9: Press and knead the Shenting Acupoint |
|  | Technique 10: Press and knead the Taixi Acupoint |

**Supplementary Table 2** Responses regarding the study implementation within the intervention group

| Items | Strongly disagree  n (%) | Disagree  n (%) | Neutral  n (%) | Agree  n (%) | Strongly agree  n (%) |
| --- | --- | --- | --- | --- | --- |
| How satisfied are you overall with the study program? | 0(0.00) | 0(0.00) | 0(0.00) | 7 (43.75) | 9 (56.25) |
| How logical does the program provided seem to you for reducing sedentary behavior (SB)? | 0(0.00) | 0(0.00) | 0(0.00) | 12(75) | 4(25) |
| How confident would you be that this program would be successful in reducing SB? | 0(0.00) | 1(6.25) | 1(6.25) | 8(50) | 6(37.5) |
| How confident would you be in recommending this program to a friend or colleague who also has SB? | 0(0.00) | 0(0.00) | 1(6.25) | 10(62.5) | 5(31.25) |
| I would feel confident doing the Dantian breathing exercise which was taught during the intervention without assistance. | 0(0.00) | 0(0.00) | 3(18.75) | 7(43.75) | 6(37.5) |
| I would feel confident doing the Baduanjin exercise which was taught during the intervention without assistance. | 0(0.00) | 1(6.25) | 3(18.75) | 7(43.75) | 5(31.25) |
| I would feel confident doing the acupressure exercise which was taught during the intervention without assistance. | 0(0.00) | 0(0.00) | 7(43.75) | 5(31.25) | 4(25) |
| I would feel confident doing the mindful stretching exercise which was taught during the intervention without assistance. | 0(0.00) | 0(0.00) | 1(6.25) | 9(56.25) | 6(37.5) |
| I would like to continue doing the Dantian breathing exercise which was taught during the intervention even after the study concludes. | 0(0.00) | 0(0.00) | 1(6.25) | 9(56.25) | 6(37.5) |
| I would like to continue doing the Baduanjin exercise which was taught during the intervention even after the study concludes. | 0(0.00) | 0(0.00) | 1(6.25) | 9(56.25) | 6(37.5) |
| I would like to continue doing the acupressure exercise which was taught during the intervention even after the study concludes. | 0(0.00) | 0(0.00) | 2(12.5) | 10(62.5) | 4(25) |
| I would like to continue doing the mindful stretching exercise which was taught during the intervention even after the study concludes. | 0(0.00) | 0(0.00) | 0(0.00) | 12(75) | 4(25) |
| Is it easy for you to understand the information provided by the instructor? | 0(0.00) | 0(0.00) | 3(18.75) | 11(68.75) | 2(12.5) |
| Do you think the daily reminders (in WhatsApp) during the experiment are appropriate? | 0(0.00) | 0(0.00) | 2(12.5) | 7(43.75) | 7(43.75) |
| Do you think the frequency of daily reminders (in WhatsApp) during the experiment is appropriate? | 0(0.00) | 0(0.00) | 1(6.25) | 8(50) | 7(43.75) |
| Do you follow the reminders to break sedentary behavior (WhatsApp reminders) for your workouts? | 1(6.25) | 4(25) | 2(12.5) | 4(25) | 5(31.25) |

**Supplementary Table 3** Participants’ Feedback in the phone interview

| **Themes** |  |  |
| --- | --- | --- |
| **Perceived Benefits from the intervention program** | Benefits for health and mood | Benefits for lifestyle change |
|  | - Enhanced the awareness of personal physical and mental health(stress, anxiety, depressive symptoms). - Minimized sedentary behavior: When tired at work, I practiced breathing techniques, acupressure, or some format of Baduanjin movements. - The training helped remind me to stretch and relax more. - Baduanjin and acupressure relieved my fatigue and eye strain from screen time. - It was a chance to calm the mind and rejuvenate the body. - Baduanjin and similar exercises benefit both mind and body, improving circulation and supporting heart and muscle health. | - The program has increased my awareness of my sedentary habits. - A timely reminder to exercise more. - I made a habit of moving every 30 minutes, even if it was just to walk around or grab something. - I tried to find time for mindful stretching or Baduanjin, whether during lunch or before bed, to relax and reduce stress. - Incorporating mindfulness into my daily routine. |
| **Challenges faced during the program** | - The 1:00-2:00 PM group practice conflicts with lunch time. - Due to late work hours, some participants may not be able to arrive at the laboratory in time for the 6:30 PM workshop. | |
| **Recommendations for the future study** | - The overall arrangement was acceptable. - Should share more videos on acupressure points. - Put the exercises in the mobile app for easy learning. | |

**Supplementary Table 4** Baseline characteristics of participants

|  |  | Mean (SD) | *t* | *p* | *Hedges’g* |
| --- | --- | --- | --- | --- | --- |
| Total sitting time (min/workday) | Intervention | 537.37(86.27) | -0.292 | 0.772 | -0.099 |
|  | Control | 547.31(109.22) |  |  |  |
| Sitting time (min/ 9h workday) | Intervention | 344.68(54.78) | -0.349 | 0.729 | -0.119 |
|  | Control | 351.47(56.72) |  |  |  |
| IPAQ | Intervention | 1529.65(763.34) | -1.616 | 0.116 | -0.541 |
|  | Control | 2240.21(1644.92) |  |  |  |
| Perceived stress | Intervention | 16.32(6.53) | -1.62 | 0.114 | -0.514 |
|  | Control | 19.79(6.69) |  |  |  |
| Hand grip(L/kg) | Intervention | 24.06(9.85) | 0.472 | 0.639 | 0.15 |
|  | Control | 22.78(6.41) |  |  |  |
| Hand grip(R/kg) | Intervention | 27.05(10.69) | 0.649 | 0.521 | 0.211 |
|  | Control | 25.31(7.47) |  |  |  |
| SBP (mmHg) | Intervention | 112.58(17.37) | 0.011 | 0.992 | 0.003 |
|  | Control | 112.53(12.99) |  |  |  |
| DBP (mmHg) | Intervention | 71.95(10.34) | 0.579 | 0.566 | 0.184 |
|  | Control | 70.26(7.33) |  |  |  |
| General self-efficacy | Intervention | 26.74(4.28) | 1.075 | 0.29 | 0.346 |
|  | Control | 25.06(5.21) |  |  |  |
| PSQI | Intervention | 5.89(3.25) | -1.137 | 0.263 | -0.361 |
|  | Control | 7.05(3.03) |  |  |  |
| Quality of life_SF-36 |  |  |  |  |  |
| Physical Component Summary (PCS)_T score | Intervention | 51.29(6.98) | 1.737 | 0.091 | -0.087 |
|  | Control | 47.17(7.64) |  |  |  |
| Mental Component Summary (MCS)_T score | Intervention | 45.52(11.42) | 1.790 | 0.082 | 0.569 |
|  | Control | 38.19(13.72) |  |  |  |

DBP=Diastolic blood pressure; IPAQ= International Physical Activity Questionnaire; PSQI=Pittsburgh Sleep Quality Index Scale; SBP=Systolic blood pressure.

Note: The sitting time data included 16 observations in the intervention group and 17 in the control group. For other outcomes, there were 19 samples in each group.

**Supplementary Table 5** Changes in sedentary behavior, physical activity, psychological and physical well-being outcomes from baseline to 4-week follow-up

|  |  | Baseline (T1) | Follow-up (T3) | Within-subject Difference | | | Change from baseline to Follow-up | Between-group Differences in Change from baseline to Follow-up | | |
| --- | --- | --- | --- | --- | --- | --- | --- | --- | --- | --- |
|  |  | Mean (SD) | Mean (SD) | t | p | Hedges’g | Mean (SD) | t | p | Hedges’g |
| Total sitting time (min/workday) | Intervention | 537.37(86.27) | 509.81(110.46) | 1.002 | 0.332 | 0.238 | -27.46(109.60) | 0.416 | 0.681 | 0.141 |
|  | Control | 547.31(109.22) | 505.39(103.80) | 1.924 | 0.072 | 0.444 | -41.92(89.83) |  |  |  |
| Sitting time (min/ 9h workday) | Intervention | 344.68(54.78) | 311.16(69.60) | 2.195 | 0.044 | 0.521 | -33.52(61.07) | -1.197 | 0.24 | -0.407 |
|  | Control | 351.47(56.72) | 345.28(76.50) | 0.367 | 0.718 | 0.089 | -6.19(69.52) |  |  |  |
| IPAQ | Intervention | 1529.65(763.34) | 2964.15(1880.41) | -3.316 | 0.004 | -0.766 | 1434.50(1783.62) | 1.628 | 0.113 | 0.545 |
|  | Control | 2240.21(1644.92) | 2559.85(2415.07) | -0.602 | 0.555 | -0.139 | 319.65(2189.62) |  |  |  |
| Perceived stress | Intervention | 16.32(6.53) | 13.11(7.87) | 2.759 | 0.013 | 0.606 | -3.21(5.07) | -0.111 | 0.912 | -0.035 |
|  | Control | 19.79(6.69) | 16.79(8.11) | 1.999 | 0.061 | 0.439 | -3.00(6.54) |  |  |  |
| Hand grip (Left/kg) | Intervention | 24.06(9.85) | 24.31(9.27) | -0.781 | 0.445 | -0.172 | 0.25(1.40) | -0.251 | 0.804 | -0.08 |
|  | Control | 22.78(6.41) | 23.26(5.42) | -0.572 | 0.574 | -0.126 | 0.47(3.61) |  |  |  |
| Hand grip (Right/kg) | Intervention | 27.05(10.69) | 26.71(9.81) | 0.692 | 0.498 | 0.152 | -0.44(2.80) | 0.132 | 0.896 | -0.042 |
|  | Control | 25.31(7.47) | 24.92(6.25) | 0.316 | 0.756 | 0.069 | -0.29(4.07) |  |  |  |
| SBP (mmHg) | Intervention | 112.58(17.37) | 110.36(16.62) | 0.757 | 0.459 | 0.166 | -1.95(11.21) | -0.062 | 0.951 | -0.02 |
|  | Control | 112.53(12.99) | 110.79(12.77) | 0.792 | 0.439 | 0.174 | -1.74(9.56) |  |  |  |
| DBP (mmHg) | Intervention | 71.95(10.34) | 72.21(11.11) | -0.165 | 0.871 | -0.036 | 0.26(6.94) | -0.371 | 0.713 | -0.118 |
|  | Control | 70.26(7.33) | 71.37(8.95) | -0.682 | 0.504 | -0.15 | 1.11(7.06) |  |  |  |
| General self-efficacy | Intervention | 26.74(4.28) | 26.26(4.58) | 0.46 | 0.651 | 0.101 | -0.47(4.49) | -0.509 | 0.614 | -0.169 |
|  | Control | 25.06(5.21) | 25.75(6.43) | -0.263 | 0.796 | -0.062 | 0.25(3.80) |  |  |  |
| PSQI_total score | Intervention | 5.89(3.25) | 5.05(3.08) | 1.757 | 0.096 | 0.386 | -0.84(2.09) | 0.397 | 0.694 | 0.126 |
|  | Control | 7.05(3.03) | 5.95(2.76) | 2.412 | 0.027 | 0.53 | -1.11(2.00) |  |  |  |
| **Quality of life_SF-36** | | | | | | | | | | |
| Physical Component Summary (PCS)_T score | Intervention | 51.29(6.98) | 52.50(5.15) | -1.363 | 0.190 | -0.299 | 1.20(3.84) | -1.028 | 0.311 | -0.326 |
|  | Control | 47.17(7.64) | 49.91(7.75) | -2.265 | 0.036 | -0.498 | 2.74(5.27) |  |  |  |
| Mental Component Summary (MCS)_T score | Intervention | 45.52(11.42) | 46.62(11.81) | -0.767 | 0.453 | -0.176 | 1.10(6.26) | -1.498 | 0.143 | -0.476 |
|  | Control | 38.19(13.72) | 43.09(12.66) | -2.342 | 0.031 | -0.515 | 4.90(9.11) |  |  |  |

DBP=Diastolic blood pressure; IPAQ= International Physical Activity Questionnaire; PSQI=Pittsburgh Sleep Quality Index Scale; SBP=Systolic blood pressure.

**Supplementary Table 6** The number of valid ActiGraph wear days during the experimental period

|  | Baseline (T1) (mean,95%CI) | Post-intervention (T2) (mean,95%CI) | Follow-up (T3)  (mean,95%CI) |
| --- | --- | --- | --- |
| Intervention | 3.13(2.48-3.77) | 3.31(2.65-3.98) | 3.19(2.66-3.71) |
| Control | 4.29(3.86-4.73) | 3.29(2.49-4.10) | 3.65(2.97-4.33) |
